# Maternal Transmission of SARS-CoV-2: Safety of Breastfeeding in Infants Born to Infected Mothers

**DOI:** 10.3389/fped.2021.738263

**Published:** 2021-12-09

**Authors:** Hayder M. Al-kuraishy, Ali I. Al-Gareeb, Francis O. Atanu, Mona A. EL-Zamkan, Hassan M. Diab, Ahmed S. Ahmed, Thabat J. Al-Maiahy, Ahmad J. Obaidullah, Sultan Alshehri, Mohammed M. Ghoniem, Gaber E. Batiha

**Affiliations:** ^1^Department of Clinical Pharmacology and Medicine, College of Medicine, AL-Mustansiriyah University, Baghdad, Iraq; ^2^Department of Biochemistry, Faculty of Natural Sciences, Kogi State University, Anyigba, Nigeria; ^3^Department of Food Hygiene and Control (Milk Hygiene), Faculty of Veterinary Medicine, South Valley University, Qena, Egypt; ^4^Department of Animal and Poultry Health and Environment, Faculty of Veterinary Medicine, South Valley University, Qena, Egypt; ^5^Department of Gynecology and Obstetrics, College of Medicine, Mustansiriyah University, Baghdad, Iraq; ^6^Department of Pharmaceutical Chemistry, College of Pharmacy, King Saud University, Riyadh, Saudi Arabia; ^7^Department of Pharmaceutics, College of Pharmacy, King Saud University, Riyadh, Saudi Arabia; ^8^Department of Pharmacy Practice, College of Pharmacy, AlMaarefa University, Ad Diriyah, Saudi Arabia; ^9^Department of Pharmacology and Therapeutics, Faculty of Veterinary Medicine, Damanhour University, Damanhour, Egypt

**Keywords:** breast milk, COVID-19, pandemic, infectious disease, maternal transmission

## Abstract

Coronavirus disease 2019 (COVID-19) is a recent epidemic disease caused by severe acute respiratory syndrome virus type 2 (SARS-CoV-2). In pregnancy, SARS-Cov-2 infection creates additional alarm due to concerns regarding the potential for transmission from the mother to the baby during both the antenatal and postpartum times. In general, breastfeeding is seldom disallowed because of infection of the mother. However, there are few exceptions with regards to certain infectious organisms with established transmission evidence from mother to infant and the link of infection of a newborn with significant morbidity and mortality. It is confirmed that pregnant women can become infected with SARS-CoV-2, although the debate on the possible vertical transmission of SARS-CoV-2 infection during pregnancy is still open. In this regard, the literature is still poor. On the contrary, the information on the safety of breastfeeding even during infections seems reassuring when the mother takes the necessary precautions. However, there are still answered questions regarding the precautions to be taken during breastfeeding by COVID-19 patients. This paper reviews the existing answers to these and many other questions. This review therefore presents a summary of the present-day understanding of infection with SARS-CoV-2 and discusses the answers around the maternal transmission of COVID-19 and the potential threat of breastfeeding to babies born to infected pregnant mothers. In conclusion, intrauterine transmission of SARS-CoV-2 infection is less likely to occur during pregnancy. Most studies suggest that COVID-19 is not transmitted through breast milk. Correspondingly, COVID-19-infected neonates might acquire the infection *via* the respiratory route because of the postnatal contact with the mother rather than during the prenatal period. International organizations encourage breastfeeding regardless of the COVID-19 status of the mother or child as long as proper hygienic and safety measures are adhered to so as to minimize the chance of infant infection by droplets and direct contact with the infected mother. Pasteurized donor human milk or infant formula as supplemental feeding can be quite beneficial in the case of mother–infant separation till breastfeeding is safe.

## Introduction

The current epidemic resulting from infection by the extreme acute respiratory syndrome coronavirus 2 (SARS-CoV-2) (a coronavirus first recognized in Wuhan, Hubei Region, China), towards, the end of year 2019, has recently affected most of the countries in the world, generating a serious impact on global health and economy (1–3). The World Health Organization (WHO) has therefore proclaimed this devastating illness as a public health epidemic of global significance (4). In pregnancy, SARS-CoV-2 infection creates additional concern regarding the potential for transmission from the mother to the baby during both the antenatal and the postpartum periods (5–7). Owing to the major alterations in the immune system of pregnant women, it has been observed that they have a greater risk of getting infected with respiratory viruses, and subsequent complications, such as severe pneumonia, may ensue following the infection (8, 9). In general, breastfeeding is seldom disallowed because of infection of the mother. However, there are few exceptions with regards to certain infectious organisms with established transmission evidence from mother to infant and the link of infection of a newborn with significant morbidity and mortality. It is confirmed that pregnant women can become infected with SARS-CoV-2, although the debate on the possible vertical transmission of SARS-CoV-2 infections during pregnancy is still open. In this regard, the literature is still poor. On the contrary, the information on the safety of breastfeeding even during infections seems reassuring when the mother takes the necessary precautions. The big question is, are there any precautions to be taken during breastfeeding in case of COVID-19 infections? This paper reviews the existing answers to these and many other questions, although there are obvious challenges on getting such answers with certainty largely as a consequence of the dearth of data on the effect of the ongoing COVID-19 pandemic on infected pregnant women, their babies, and the entire population of children. This review therefore presents a summary of the present-day understanding of infection with SARS-CoV-2 and discusses the answers around the maternal transmission of COVID-19 and the potential threat of breastfeeding to babies born to infected pregnant mothers.

## The History and Morphology of SARS-CoV-2

**Figure 1:**
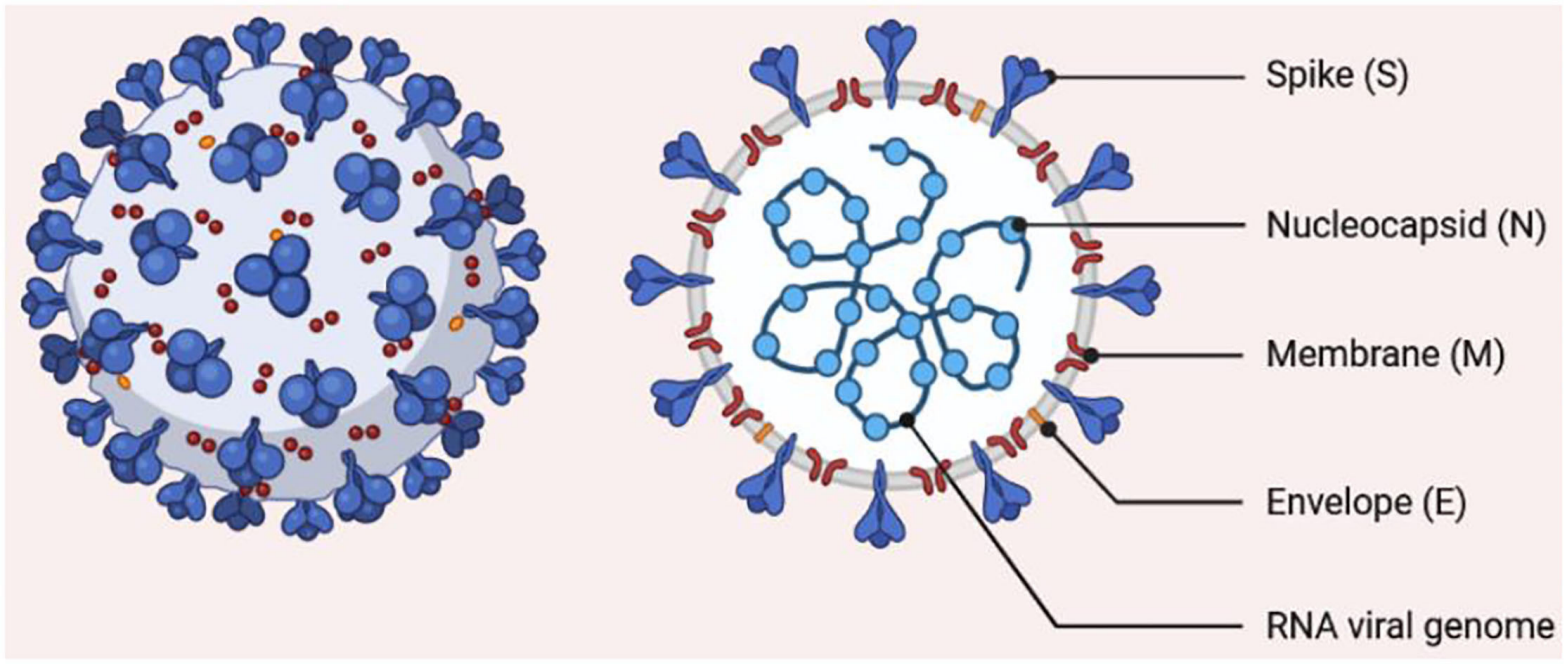
Morphology of SARS-CoV-2. The structure shows a single-stranded RNA encapsulated by a membrane carrying spike proteins.

In December 2019, the number of individuals presenting with fever and/or dry cough with normal or reduced lymphocyte count and who were at first suspected to have “fever of unknown origin with pneumonia” kept on rising in Wuhan (10). The organism responsible for this mysterious pneumonia was later isolated and named SARS-CoV-2. The virus was associated with a widespread human-to-human transmission that caused a serious respiratory distress and high mortality rate (11). SARS-CoV-2 is a single-stranded RNA virus with a crown-like appearance, belonging to Sarbecovirus of the genus *Betacoronavirus*. The particles of this virus possess spikes and are enveloped, spherical, oval, or pleomorphic, with an estimated size of 60 to 140 nm (Figure 1). The gene identity of the SARS-CoV-2 genome is roughly 80% compared with other coronaviruses recently blamed for global public health issues (SARS-CoV and Middle East respiratory syndrome virus, MERS-CoV) (12). SARS-CoV-2 also shares sequence similarity with the Guangdong pangolin coronaviruses in the receptor-binding domain, which suggest that pangolins may serve as an intermediate host of the virus before spreading to human beings (13).

## COVID-19 Transmission

**Figure 2:**
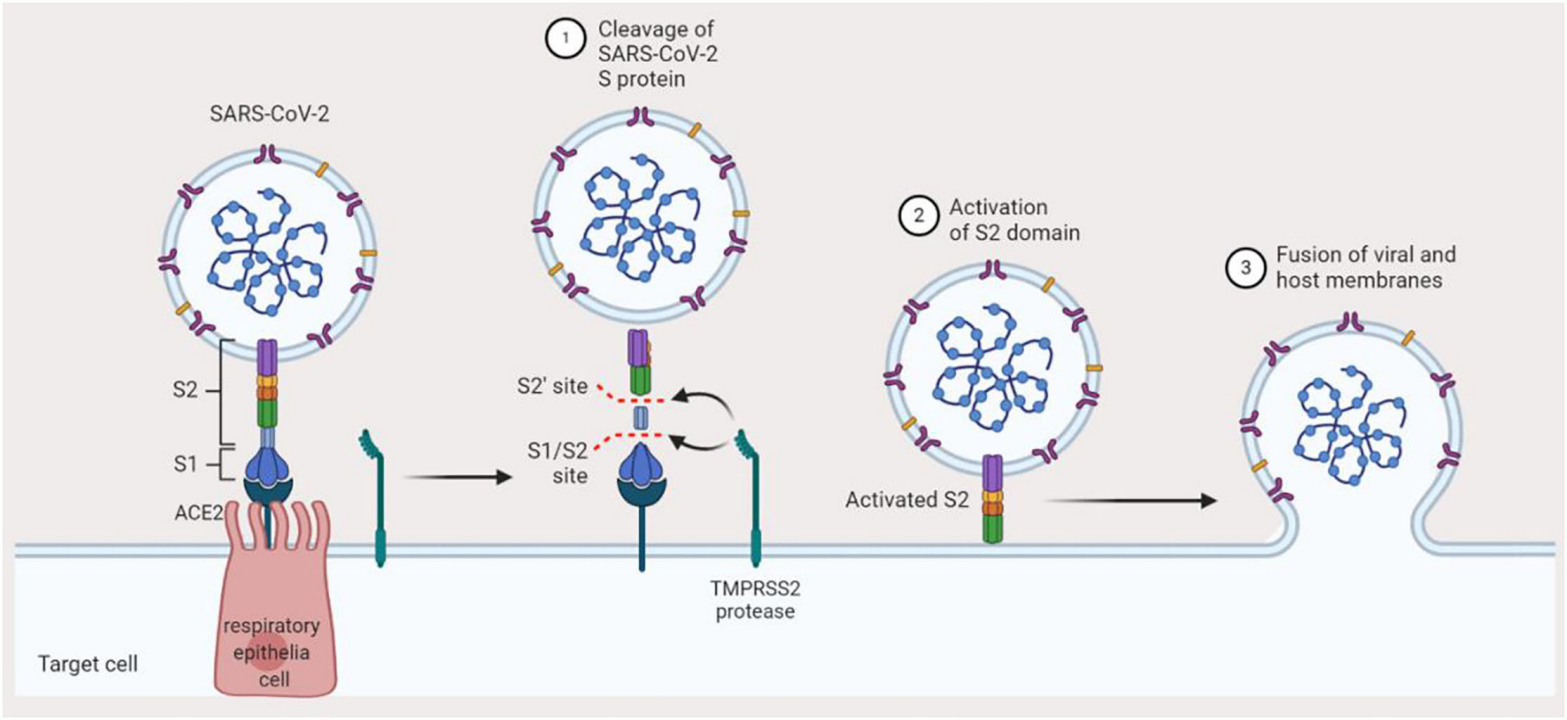
Mechanism of SARS-CoV-2 infection. Binding of SARS-CoV-2 to angiotensin-converting enzyme-2 in the host preludes the invasion of the host cell. The TMPRSS2 protease of the host cleaves the S1/S2 site of the spike protein, which activates the S2 domain of the spike protein. Finally, the virus gets fused to the host membrane and hence invades the cell.

Although COVID-19-positive individuals who are symptomatic constitute those that mostly transmit the disease, the risk of transmission by asymptomatic individuals cannot also be neglected. Epidemiologically, COVID-19 shares some similarities with SARS and MERS, i.e., it is mainly transmitted *via* aerosols or droplets and physical contact with infected individuals (14). There is also a likelihood of feco-oral transmission since the nucleic acid of SARS-CoV-2 was found in the feces of infected patients in the US and China (15). However, mother to child vertical transmission is still a matter of debate (so far, there has been no recorded evidence of SARS and MERS intrauterine vertical transmission) (16, 17). Although the death rate associated with SARS-CoV-2 tends to be relatively lower in comparison with SARS and MERS, it still poses a great deal of threat on the global community owing to its greater infectious capability and its easy spread (18). A significant proportion of the community, especially breastfeeding mothers, is affected by this threat. Following exposure to SARS-CoV-2, the viral particle binds to angiotensin-converting enzyme 2 (ACE2) of the host cell in the respiratory system. There is a major expression of ACE2 in the alveoli, trachea, bronchi, and bronchial serous gland epithelium (19). Following entry into the host cell, SARS-CoV-2 undergoes replication, and the newly formed virions are subsequently released and infect more cells (Figure 2). To date, no definitive antiviral agent has been found for the cure of COVID-19. However, the four authorized vaccines (Comirnaty—Pfizer-BioNTech, Spikevax—Moderna, Vaxzevria—Oxford-AstraZeneca, and Janssen—Johnson & Johnson) have been thoroughly tested and found to be safe and effective in preventing severe COVID-19. The major treatment offered is symptomatic treatment by using oxygen therapy for patients with a life-threatening disease. Pharmacological approach may also be applied to treat acute uncomplicated cases. However, more treatment options are required, thus necessitating the search for newer therapeutic agents (20, 21). Presently, the WHO and other stakeholders are faced with the task to discover effective therapeutic agents to combat SARS-CoV-2 or make possible the fast development of vaccine(s) that prevent the disease. With the lack of an effective medicine and sufficient vaccine doses for the entire world population, providing effective monitoring of the infection source, early diagnosis, and prompt and efficient supportive management of COVID-19-positive patients are the best methods at present. At a personal level, good hygiene and avoidance of massive gathering will play a crucial role in preventing the disease.

## Clinical Symptoms of COVID-19

**Figure 3:**
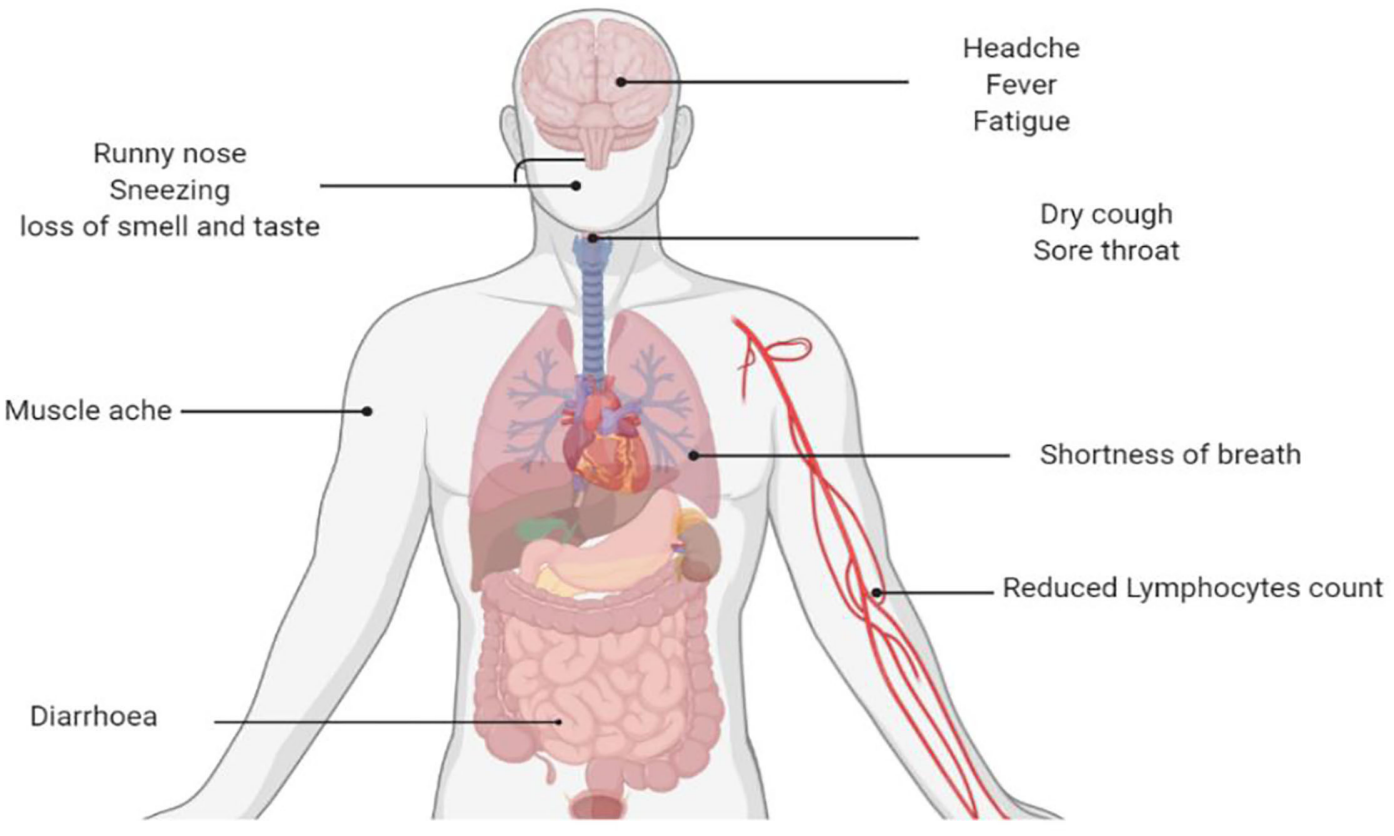
Clinical symptoms of COVID-19 infection.

The new coronavirus linked with severe respiratory syndrome in central China's Wuhan City was named COVID-19 by WHO on January 10, 2020 (22). It may present with symptoms of respiratory diseases and can also involve the gastrointestinal tract, liver, and nervous system with significant outcomes (Figure 3) (23). Although much emphasis has been given to susceptible individuals, principally the aged and people with coexisting ailments, pregnant mothers, and their babies may also constitute a high-risk group. Till today, there is still a dearth of reported literature particularly on case series and case reports concerning infection by SARS-CoV-2 in pregnancy, risk of mother–child, and neonatal infection. Interestingly, SARS-CoV-2 infection in newborn babies has been reported to be comparatively milder (24). Most children with COVID-19 experience mild symptoms and recovered within a week; however, a small proportion remain un-well for more than 4 weeks (25). Liu et al. (26) revealed a guideline for the management of children with COVID-19. Most of children with COVID-19 are asymptomatic compared to adults. A computed tomography scan should not be used for the diagnosis of COVID-19, and there is no evidence for the effectiveness of corticosteroids and antimicrobial and antiviral drugs in children with COVID-19.

COVID-19 presents in a variety of ways, ranging from asymptomatic form, minor respiratory symptoms, to severe lower respiratory infection associated with respiratory failure, leading to hospitalization of a significant number of infected individuals that often necessitates intensive care (27).

The incubation period (IP) may vary according to the severity of the disease and is dependent on the age and immunologic reaction of the individual. Elderly individuals above the age of 70 tend to have a shorter IP of 11 and a half (11.5) days compared to patients <70 years (20 days) (28). COVID-19 patients may present with common cold and influenza-like symptoms that are not complicated. However, individuals with coexisting diseases like diabetes mellitus, cardiovascular diseases, pulmonary diseases as well as other chronic disorders may present with more severe symptoms. In such cases, the infection can become overwhelming that may occasionally result in death. Some individuals may also remain asymptomatic. Based on a recent definition by the WHO, the most frequent symptoms of COVID-19 include the following: fever, dry cough and sore throat, fatigue, and diarrhea (26, 29). In addition, serious shortness of breath was seen in approximately 20% of patients, and about 13% presented with sore throat or severe headache. By and large, SARS-CoV-2 has a preponderance for the pulmonary tract. This makes some patients highly susceptible to a variety of complications, particularly acute respiratory distress syndrome and acute cardiac damage (30). The initial target of the virus is the respiratory epithelial lining, where it takes over the machinery of the cells to replicate into multiple copies with a further spread to the surrounding cells. The virus triggers an immune reaction that causes constitutional symptoms such as high-grade fever and generalized malaise. However, the immune response becomes disrupted, leading to an exaggeration of inflammatory response that is not wanted by the body. Recent findings also suggest that SARS-CoV-2 infection can cause gastrointestinal (GI) symptoms such as anorexia, diarrhea, and emesis. Individuals who presented with COVID-19-related GI symptoms exhibited a tendency for complications compared to those without the GI symptoms. The pathophysiology of this disorder is believed to be a result of the destruction of the GI bacteria by SARS-CoV-2 (31).

## Is COVID-19 Infection Vertically Transmitted During Pregnancy?

The vertical transmission of COVID-19 from the mother to the fetus or newborn is supported by a drought of evidence. However, out of 222 newborns, whose mothers were suspected or confirmed to be SARS-CoV-2 positive, perinatally or neonatally admitted to hospitals with infection/pneumonia as reported in 20 studies, only 13 COVID-19-positive newborns were identified as positive for SARS-CoV-2 (32–34), and those were confirmed within 36 h to 17 days post-delivery. Dong et al. (35) and Zeng et al. (36) speculated a mother-to-child transmission, as they reported elevated values of IgG and/or IgM to SARS-CoV-2 in neonates of COVID-19 mothers. The evidence was not conclusive as the RTPCR-RNA results of maternal or neonatal samples, *e*.*g*., infant swabs or amniotic fluid, as a confirmation, were lacking in the case of Dong et al. (35) and was negative for neonatal serum and throat swabs in the study done by Zeng et al. (36). To date, it is uncertain whether the vertical transmission of SARS-CoV-2 infection during pregnancy occurs or not, though cases of neonates with a positive nasopharyngeal swab after birth could be connected to immediate horizontal transmission postpartum due to contact with the infected mother or with infected healthcare personnel at the time of delivery (37). However, many studies revealed that transplacental transmission is possible and more likely occurs in the last weeks of pregnancy. It has been shown that neonatal viremia is linked with SARS-CoV-2 placental infection, and the delivered neonates may develop severe symptoms similar to those of infected adults (38, 39).

Furthermore, a multidisciplinary group from WHO proposed a simple classification system about the vertical transmission of SARS-CoV-2 to permit for the comparison of data from various studies and to appreciate the clinical consequences and concerns for neonates born to infected mothers (40).

In fact, on the basis of RT-PCR test results of neonatal samples, but not IgM identification with its high frequency of false positive and false negative results, evidence to support *utero* transmission could be derived (35, 36).

Three neonates born to a woman with COVID-19 were likewise diagnosed as positive for SARS-CoV-2 by qRT-PCR (41). However, as noted in the study, samples of placenta, amniotic fluid, or cord blood to detect SARS-CoV-2, which is crucial for affirming that the SARS-CoV-2 infection in the neonate was due to intrauterine transmission, were lacking; moreover, the neonatal throat swab was collected at more than 48 h after delivery. The nucleic acid of the virus has also been detected in blood samples of newborns (36), but as explained by Wang et al. (42), nucleic acids do not represent infectious particles, plus the possibility of a false positive and a false negative result of the IgM which was the only detected one.

Despite the documented usage of preventive measures before and after birth, some neonates were positive for COVID-19 (26, 30–33), but even in these cases, there was no proof that vertical transmission occurred. In contrast, Martins-Filho et al. (43) stated that biological samples collected immediately after birth from the upper respiratory tract (throat or nasopharyngeal swabs) of neonates and placental tissues showed negative results for the presence SARS-CoV-2 by RT-PCR test.

Neonate specimens obtained within 24 h after birth also tested negative (41, 44–47), yet there are occasional cases of SARS-CoV-2-infected newborns. Vertical transmission from the mother to the neonate is uncertain; particularly the existence of SARS-CoV-2 in the placenta, cord blood, amniotic fluid, breast milk, or neonatal throat swab could not be demonstrated using real time-PCR (42, 43, 45–48). Furthermore, the intrauterine vertical transmission of SARS and MERS to any neonate could not be supported by any evidence (16, 17). Accordingly, to date, there is no proof to support vertical transmission *in utero*, and the transmission may likely occur during the delivery of the babies (49–51) or through horizontal contamination by droplets between the mother and the newborn. The WHO states that whenever a mother is severely ill due to COVID-19 or if other complications preclude her from taking care of and/or breastfeeding her infant, she should be supported to safely express breast milk and deliver it to her baby (52, 53).

## Health Benefits of Human Breast Milk

The positive benefits of breastfeeding have been well recorded for both newborns and mothers since it is healthy and clean and includes antibodies that serve as a protection against many common childhood diseases. The WHO declares breast milk as the best food for babies (54). Breast milk provides the nutrients and energy needed during the first months of life of all children and proceeds to supply up to half or more of the nutritional needs of a child during the second half of the first year and up to one third during the second year of life. Furthermore, breastfeeding women are reported to have a significantly reduced risk of developing chronic diseases like hypertension, diabetes, and breast cancer (55).

Breastfeeding is therefore considered very important by most families because of its ability to improve the well-being of both the mother and the baby, and as such a societal value is attached to it (56, 57). Prompt commencement of breastfeeding is associated with a significant protection of infants from getting infected and a reduction in infant mortality (58), particularly in premature babies (59). The WHO recommendation is that breastfeeding should commence within the first hour of birth and continued till the age of 2 years. However, this recommendation faces a lot of challenges when the mother suffers from infectious diseases.

Colostrum (the first nutrient-rich breast milk made in the first few days of birth), apart from providing nourishments, is rich in enzymes like lysozyme and lactoferrin, immunoglobulins, cytokines, and many hormones, all of which work in synergy to provide passive immunity to the baby and stimulate further improvement in the immune system of the newborn (60). The antimicrobial elements found in colostrum and breast milk share some prominent features, such as the ability to resist degradation by GI enzymes, and are also capable of protecting the mucosal linings and the destruction of bacteria without causing an inflammation (61). The recommendation of exclusive breastfeeding (which refers to the exclusive feeding of babies with breast milk only without any other food or drink within the first hour of birth until 6 months of age) is therefore justified by the presence of these important elements (62). Similarly, the presence of immunomodulatory proteins in human breast milk plays a significant role in protecting the babies from viral diseases such as respiratory tract infections and GI disorders (63). Recently, a number of researchers investigated the potential protective roles of breastfeeding against susceptibility to common ailments whose pathophysiology is associated with inflammatory responses. A significant amount of epidemiological and experimental data investigating the influence of breast milk against many organisms causing respiratory and GI disorders is available. The investigations revealed that human breast milk is rich in antibodies against *Salmonella typhimurium, Shigella, Vibrio cholerae, Campylobacter, Streptococcus pneumoniae, Bordetella pertussis, Haemophilus influenzae*, and HIV respiratory syncytial virus and other pathogens (64, 65). Interestingly, an epidemiological study has suggested that babies that were exclusively breastfed have greater protection against a range of infectious diseases (66).

## Breast Milk Antibodies and Potential for SARS-CoV-2 Reactivity

**Figure 4:**
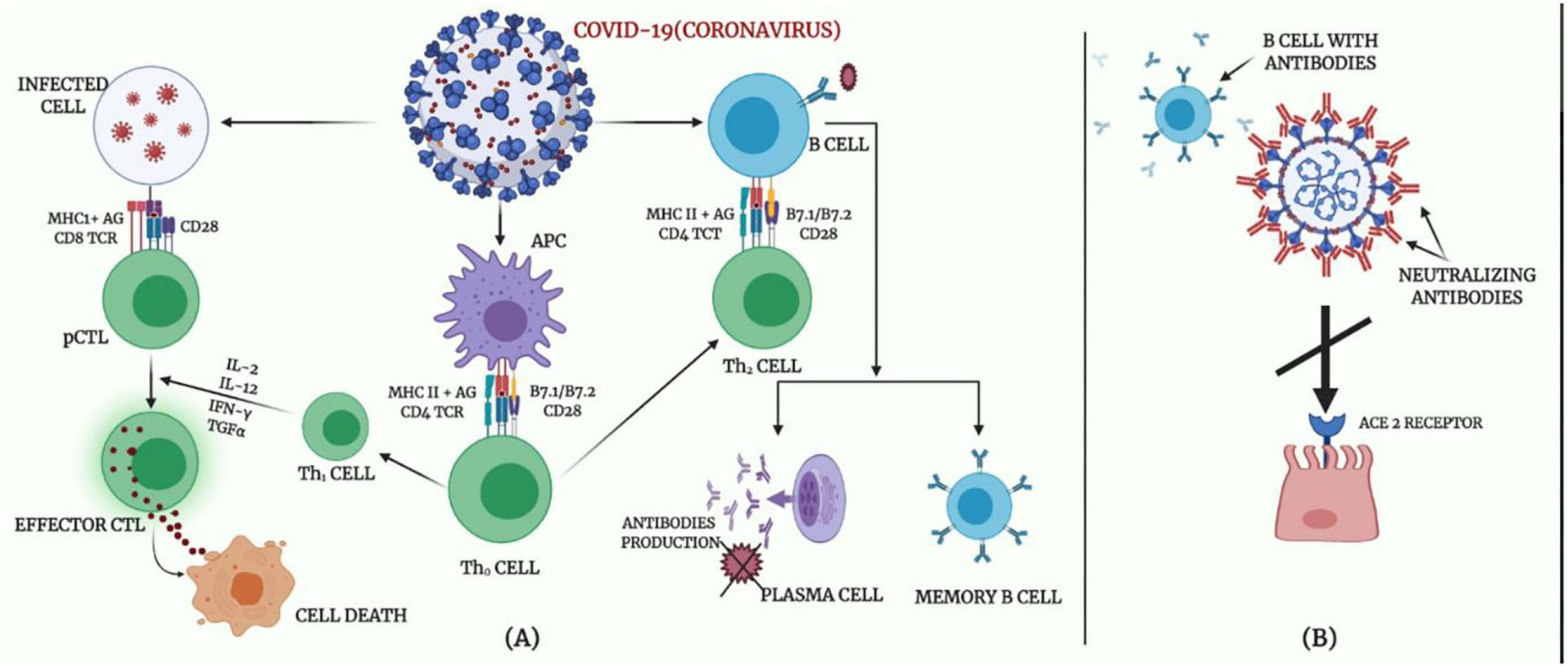
Body immune response to COVID-19. **(A)** Adaptive immune response against coronavirus. **(B)** Proposed mechanisms of neutralizing antibodies in COVID-19 infection.

The immune system is the superior defense against COVID-19 since it maintains the innate capacity of the body to defend against viruses and resist infections, especially in the absence of a vaccine with proven efficacy against SARS-CoV-2 transmission, even for infants and children. Innate immunity (rapid response), adaptive immunity (slow response), and passive immunity constitute the immune defense of the body. Passive immunity is that obtained with the transfer of immunoglobulins naturally, such as through the placenta or breast milk, or prophylactically, such as by administering immunoglobulins intravenously. Active immunity is that which develops when the individual actively produces his own antibodies, naturally during an infection or as prevention induced by vaccination procedures. The immune system functions appropriately when the body is infected by any infectious agent for the first time, (67); however, exaggerated immune response may induce tissue damage as in the current situation of COVID-19. When the immune system cells become well learned, they complete their jobs in fighting the infection, and the B cells are differentiated into plasma cells, by the support of T cells, which afterwards express specific antibodies to a viral antigen. An efficient neutralizing antibody completely blocks the virus from invading the host cells to suppress the infection and basically plays a very strong defensive role at the later stage of infection and prevents an infection from recurrence (Figure 4).

Several antibodies associated with human breast milk protect against the invasion of microorganisms and pathogenic substances, including viral infection. These antibodies build up immunological memories as the mother comes in contact with different pathogenic substances throughout her life (68). These antibodies form a major part of the protein content of the colostrum secreted in the first few days of the lactation period. The concentrations of these antibodies decrease as the lactation progresses. However, the number of immunoglobulins received by the child is the same throughout the lactation period as a result of increased milk intake (63). The most important immunoglobulin present in colostrum and breast milk is secretory IgA (SIgA) (69). The SIgA antibodies are most relevant in the mucous membrane where they play a defensive role. In this membrane, they prevent the entry of microorganisms into the tissues as well as inflammation from taking place while consuming less energy (70). Therefore, interactions between human milk IgG and SARS-CoV-2-reactive antibody might be occurring.

IgG constitutes only ~2% of milk antibodies (71); even though the strength of the milk immune reaction to SARS-CoV-2 is still unclear, they seem to be partially spared by severe COVID-19 infection. However, they can be partially responsible for spreading the virus (72, 73). More importantly, the milk Abs, especially the secretory class which has been shown to possess high resistance to proteolytic degradation in the respiratory tissues, could be purified and used as COVID-19 therapeutics (74, 75). In a preliminary report by Lee et al. (76), they tested the reactivity of antibodies to the receptor binding domain of COVID-19 Spike proteins using ELISA kits for IgA, IgM, IgG, and secretory Ab, comparing 15 milk samples from previously infected patients with SARS-CoV-2 and 10 negative control samples that were obtained before December 2019. It was found that 80% of the samples showed reactions with IgA, while all the samples were positive for the reactivity of secretory Ab, which suggests that the IgA is mainly SIgA. The mean of COVID-19 group optical density value of pure milk was substantially greater for secretory-type Abs (*p* < 0.0001), IgA (*p* < 0.0001), and IgG (*p* = 0.017), but not for IgM compared with the mean value of the pre-pandemic community. Conclusively, it is indicative from these data that a potent SIgA-dominant SARS-CoV-2 immune response exists in the majority of mother's milk after an infection. However, studies are recommended to comprehensively explain this response. The IgM antibodies, at concentrations of around 2.5 mg/ml, are the second most concentrated immunoglobulin in human colostrum. The high affinity of IgM antibodies to react with pathogens may help to protect the mucosal surfaces of the infants. The concentration of the IgG is low in the human milk, with a concentration of about 0.1 mg/ml, accounting for just 10% of its serum values. It possesses an opsonization activity for activating antibody-dependent cytotoxicity and the complement system in addition to its neutralizing activity (77). The SIgA is responsible for the majority (80 to 90%) of the total immunoglobulins in human milk, and any infant that is fed exclusively on breast milk will receive about 0.3 g/kg of the protein per day. Meanwhile, it acts locally because only 10% of the antibody is absorbed in the intestines and transported to the bloodstream (78, 79).

## Is Breast Milk Produced by a COVID-19-Infected Mother Safe for a Newborn?

Before making a decision to proceed with breastfeeding or otherwise, the SARS-CoV-2 virus must be detected in the colostrum and breast milk of a nursing mother who is COVID-19-positive in order to balance the potential positive impacts of breastfeeding against the predicted risk that the newborn will contract the infection during breastfeeding. Breastfeeding is one of the proposed routes of mother-to-child transmission of SARS-CoV-2. The existing data are limited and are not enough to confirm the vertical transmission of SARS-CoV-2 *via* the breast milk. Chen et al. (80) and Martins-Filho et al. (43) reported that breast milk samples taken from mothers with COVID-19 pneumonia were negative for COVID-19; moreover, Duran et al. (81) reported that studies of the conducted systematic review that examined samples of breast milk were negative for the presence of SARS-CoV-2. Pereira et al. (82) also stated that no neonates got an infection during breastfeeding from 22 COVID-19-infected mothers. On the other hand, just three out of 46 breast milk samples obtained from mothers who had COVID-19 tested positive for viral particles by RT-PCR, and only one infant among the three that received positive breast milk for viral RNA particles tested positive for COVID-19, yet infant nursing practices were not registered (83). In addition, SARS-CoV-2 seems to spare human breast milk, and horizontal transmission from mother to neonate might occur through respiratory droplets rather than through breast milk (84). Olivine et al. revealed that the affected neonates are mostly pauci-symptomatic or asymptomatic, and a minority of them presented with severe presentations (85). Moreover, a substantial uncertainty remains concerning whether breast milk is able to transmit SARS-CoV-2 from a mother to her neonate. Human milk from an infected mother with SARS-CoV-2 is the potential source of anti-SARS-CoV-2 IgA and IgG that have a neutralizing activity against SARS-CoV-2 (86). These findings suggest continuing breastfeeding during mild to moderate COVID-19, as breast milk provides specific immunological benefits to the neonate of an infected mother (87).

The route through which this infant with COVID-19 got the infection is unknown, whether it was through the milk from the infected mother or from her droplets during breastfeeding due to the close connection. However, it is possible that infants who receive breast milk from these infected mothers gain some level of immunity and protection predicated on the idea that breast milk would provide the babies with antibodies and anti-infective factors that save neonates from acquiring infections. One of the forms in which breastfeeding saves babies from illness and mortality is the occurrence of IgA in breast milk. In the breast milk of mothers previously infected with COVID-19, IgA antibodies with COVID-19 reactivity have been confirmed, but their intensity and durability have not been sufficiently evaluated to emphasize the defense against COVID-19 among breastfed infants (83). Thus, the recommendation of most international associations is therefore to support the use of breast milk from either COVID-19 confirmed or unconfirmed cases (87–92).

These data suggest that SARS-CoV-2 may not be transmitted through breast milk. Based on these results, it is illustrated that most guidelines on neonates do not prohibit mothers with COVID-19 from breastfeeding. However, precautions, such as mask wearing, and other hygiene procedures must be observed when breastfeeding.

## Precautions and Suggestion by Organizations on Breastfeeding for a COVID-19-Positive Mother

It is well recognized that the best form of nutrition that a neonate or infant can get is from breast milk (93). Numerous benefits and values are associated its nutrients for the baby, like providing passive immunity for the baby against various infections (67). Throughout this COVID-19 pandemic, the most influential associations, such as WHO, UNICEF, RCOG, ISUOG, and ABM, recommend breastfeeding but with the application of strict protective measures. Additionally, they often enhance skin-to-skin care as possible, particularly after birth, to assist their adaptation to the external environment and breastfeeding stimulation. Proper precautions and guidelines should be followed to guarantee that the secreted breast milk is not contaminated. It is important to carefully collect the breast milk with various collection techniques by using a dedicated breast pump in order to prevent contamination (84). SARS-CoV-2 virus has not been detected in human milk samples in most reports (94), and although SARS-CoV-2 RNA is detected in some human milk samples, the risk of milk contamination by maternal respiratory secretions could not be ruled out (94). Besides these, Groß et al. (95) observed that neonate SARS-CoV-2 infection may occur by the breast milk of mothers with COVID-19; other modes of transmission remain unclear. Above all, the presence of viral particles does not imply the possibility of transmitting the infection *via* breastfeeding. Therefore, studies of milk samples from COVID-19-infected lactating mothers and possible virus transmission through breastfeeding are needed to build up recommendations on whether mothers with COVID-19 should breastfeed.

It has been recommended by the WHO and UNICEF that, by following infection prevention precautions, the newborn should be breastfed within the first hour of birth by using the standard breastfeeding guidelines (85, 86). The staff receiving the milk should also ensure that the milk is safe by following precautionary measures (89). It remains unclear whether SARS-CoV-2 virus can be infiltrated into breast milk and transmitted to a child through breastfeeding. Different precautions and suggestions have been made by different organizations about breastfeeding by a COVID-19-infected mother (shown in Table 1).

**Table 1:** Precautions and suggestion by organizations on breastfeeding by an infected mother.

**Organization**	**Direction on the prevention of mother-to-child transmission**	**Reason(s)**	**References**
Chinese Pediatrics COVID-19 Working Group	Feeding of infants with formula or donor breast milk should be after the formula or milk has been isolated in the appropriate unit for a period of 14 days	No obvious reasons were provided	Wang et al. (90)
World Health Organization (WHO)	Mothers with health issues should be allowed to collect the breast milk to feed her child. Milk from the human milk bank could replace mother's milk in case she is unable to lactate	WHO advises that breastfeeding should be based on the willingness of the mother as well as her family and the advice of her health provider However, to avoid transmission to the infant, all protective practicable measures must be taken	WHO (85)
United Nations Children's Fund (UNICEF)	The nursing mother should continue to breastfeed while taking all precautionary measures	In order to prevent the separation of the neonate from the mother	UNICEF (86)
Centers for Disease Control and Prevention (CDC)	Infant formula is the first feeding choice in order to keep the baby safe if the mother is either suspected or positive with COVID-19 However, if they are kept together, preventive measures should be taken to prevent the baby from contracting the virus	It is important to follow safety procedures to prevent the contraction of the virus by the baby	CDC (84)
Royal College of Obstetricians and Gynecologists (RCOG)	The mother and the baby should be together. However, there should be a sufficient justification to keep the mother apart from her baby in case of necessity	It is believed that breastfeeding is essential for the newborn. In addition, the benefits outweigh the adverse effects that may be associated	RCOG (91)
International Society of Ultrasound in Obstetrics and Gynecology (ISUOG)	As long as the mother is not badly affected by the infection, rooming in and breastfeeding can be achieved	No justification provided	Poon et al. (92)
Italian National Institute of Health (ISS)	Either in case of suspicious or confirmed infection, the mother should directly breastfeed or express her breast milk if she is ready to do so	Since breastfeeding is important, preventive measures must be taken to minimize the chance of transmission of the virus	ISS cited by Davanzo et al. (87, 93)
Academy of Breastfeeding Medicine (ABM)	The management of the hospital should determine whether to room in the suspected or confirmed mother with her baby or separate them	A safe procedure should be adopted	ABM (94)

## Sources of Breast Milk During the COVID-19 Pandemic

During the COVID-19 pandemic, the availability of infant nutrition and care interventions like parental assistance and breastfeeding is still a troublesome problem. Efforts are ongoing globally to overcome those problems. The WHO has repeatedly recommended that mothers who are too sick to practice breastfeeding should offer their expressed breast milk (85). Guidelines have also been issued by the Indian Council of Medical Research, and other professional societies endorse a similar recommendation (96–98). Pasteurized donor human milk (PDHM) offered by human milk banks (HMB) is preferred over milk formula in the case of disability to breastfeed, and as soon as mothers get better, they should be encouraged to resume breastfeeding (85–97). Compared to formula milk, PDHM is better at lowering the likelihood of necrotizing enterocolitis, sepsis, diarrhea and feeding intolerance, and duration of stay in neonatal intensive care units (98). Supplementary PDHM feeding is related to the increase in breastfeeding at the age of 6 months of life (99). As reported by the National Guidelines for Lactation Management in Public Health Facilities (100), it is suggested that facility-based lactation management centers should be established at all levels of the public health system like comprehensive lactation management centers (CLMCs) or integrated milk banks at tertiary centers, lactation management units at secondary centers, and lactation support units at primary care centers (100). However, universal screening of breast milk for SARS-CoV-2 is not necessary (84).

### Importance of Human Milk Banking Services During the COVID-19 Pandemic

Human milk banking services have been temporarily suspended in many centers as a result of the imposed lockdown, which led to irregularity and absenteeism of staff. Owing to early discharges and the cancellation of outpatient facilities, the number of facility-based milk donors has declined. During the lockdown, most home and community collection of donor milk has been cut off. Moreover, some mothers may be unwilling to donate for fear of donating as an exposure point for infection.

Dedicating time to milk culture is difficult for a few hospitals, while outsourcing has become difficult to accomplish for many due to restrictions on transportation amid the lockdown. Some units retain PDHM for the most ill, needy, and weak babies in order to reimburse the decreased supply, which, in turn, may cause an increase in feeding only formula for babies without mother's milk. PDHM supplied at any COVID-19 breakup should be banded together with optimum lactation assistance in order to assure the supply of mother's milk as soon as possible, minimizing the PDHM from being excessively demanded so that it can be provided to any of those babies who are in desperate need of it. Greater caution and adjustment of donor screening procedures should be applied as suggested in the guidelines of professional bodies (97). Staff of comprehensive lactation management centers should be inquiring about the history of travel and contact, and symptomatic/at-risk donors are not liable to donate their milk. Strict hygienic and sanitary protocols are also implemented during milk expression, handling, and transport. Regardless that the external laboratory facilities are limited, few CLMCs collect and store donated milk so it can be thoroughly examined as soon as laboratory facilities are accessible.

### Processing of Donor Human Milk

Mother's own milk (MOM) is the first choice of feeding for babies, including prematurely born ones. However, when the MOM is insufficient (most often in NICU), the donor human milk (DHM) collected from well-established banks of human milk is the milk of choice for those babies. A clearly stated statement from the WHO/UNICEF indicates that: “HMBs should be made available in appropriate situations” (101). Additionally, pasteurization of such DHM supplied to HMBs must be performed to destroy any microbial agents, including bacteriological and viral agents, and make it safe (102). The ideal process of pasteurization has a rapid heating phase, constant temperature phase, and a final rapid cooling phase (101). Currently, the most internationally recommended pasteurization method in the guidelines for the management of HMBs is the Holder pasteurization method. In this method, the pasteurization process is performed at 62.5°C for 30 min (84, 103). There is retention of majority of the protective and beneficial components in the pasteurized human milk (HM) (84). However, some of the missing substances may include some HM nutritionally and biologically important properties that may eliminate the useful microbiota of the fresh HM and its bacteriostatic properties, thus rendering the milk more liable to contamination after heat treatment. It also decreases the nutritional value of the milk (84).

The major limitation to the use of DHM for preterm infants is the loss of some biologically active substances, including immunological components, mainly due to the Holder pasteurization process (104, 105). Despite that Holder pasteurization causes loss of biologically active components, other components, including glucose, oligosaccharides, vitamins like vitamins A, E, D, and B_12_, gangliosides, lactose, cytokines, folic acid, and growth factors are maintained (106). Numerous devices are used to perform pasteurization. Despite the different devices, however, the most common source of heat for the pasteurization process is hot water; however, in some devices, hot water is replaced by moving hot air. The latter pattern of pasteurization is different from that of hot water pasteurization (107).

In the COVID-19 era, studies regarding the effects of anti-COVID-19 vaccines on breastfeeding are prerequisite to illustrate whether maternal vaccination results in the secretion of SARS-CoV-2 antibodies into breast milk and any potential adverse effects among women and their neonates (108). It has been shown that anti-SARS-CoV-2 antibodies in the breast milk following vaccination have strong neutralizing effects on SARS-CoV-2, suggesting a potential protective effect against infection in the neonates and infants (108). Taken together, anti-COVID-19 vaccines may increase the protective value of breast milk against SARS-CoV-2 infection.

## Conclusion

In conclusion, intrauterine transmission of SARS-CoV-2 infection is less likely to occur during pregnancy, such as SARS-CoV during the Asian epidemic (109). Most studies clarified that COVID-19 is not transmitted through breast milk. Correspondingly, COVID-19-infected neonates might have acquired the infection *via* the respiratory route because of postnatal contact with the mother rather than during the prenatal period (110). Regardless of the COVID-19 pandemic, international organizations keep on boosting breastfeeding as it is safe as long as strict proper hygienic and safety measures is achieved to minimize the hazard of infant infection by droplets and direct contact with the infected mother (111). Pasteurized donor human milk or infant formula as supplemental feeding can be quite beneficial in the case of mother–infant separation till breastfeeding is safe (112–114).

## Author Contributions

HA-k, GB, FA, ME-Z, HD, SA, and AA-G contributed to the conceptualization of this study. HA-k, AO, and FA formulated the methodology. FA, AO, MG, and AA took charge of the software. HA-k, SA, MG, HD, AA, and GB performed the validation and took charge of the resources. HA-k, FA, GB, AO, HD, AA, and TA-M contributed to writing of the original draft preparation. FA, ME, MG HD, AO, and AA reviewed and edited the manuscript draft. HA-k, TA-M, and GB were in charge of supervision and project administration. All authors have read and agreed to the published version of the manuscript.

## Conflict of Interest

The authors declare that the research was conducted in the absence of any commercial or financial relationships that could be construed as a potential conflict of interest.

## Publisher's Note

All claims expressed in this article are solely those of the authors and do not necessarily represent those of their affiliated organizations, or those of the publisher, the editors and the reviewers. Any product that may be evaluated in this article, or claim that may be made by its manufacturer, is not guaranteed or endorsed by the publisher.
